# 11Beta‐hydroxysteroid dehydrogenase‐1 deficiency or inhibition enhances hepatic myofibroblast activation in murine liver fibrosis

**DOI:** 10.1002/hep.29734

**Published:** 2018-02-22

**Authors:** Xiantong Zou, Prakash Ramachandran, Timothy J. Kendall, Antonella Pellicoro, Elena Dora, Rebecca L. Aucott, Kajal Manwani, Tak Yung Man, Karen E. Chapman, Neil C. Henderson, Stuart J. Forbes, Scott P. Webster, John P. Iredale, Brian R. Walker, Zoi Michailidou

**Affiliations:** ^1^ BHF Centre for Cardiovascular Science The University of Edinburgh Edinburgh UK; ^2^ MRC Centre for Inflammation Research The University of Edinburgh Edinburgh UK; ^3^ MRC Centre for Regenerative Medicine Queen's Medical Research Institute Edinburgh UK

## Abstract

A hallmark of chronic liver injury is fibrosis, with accumulation of extracellular matrix orchestrated by activated hepatic stellate cells (HSCs). Glucocorticoids limit HSC activation *in vitro*, and tissue glucocorticoid levels are amplified by 11beta‐hydroxysteroid dehydrogenase‐1 (11βHSD1). Although 11βHSD1 inhibitors have been developed for type 2 diabetes mellitus and improve diet‐induced fatty liver in various mouse models, effects on the progression and/or resolution of liver injury and consequent fibrosis have not been characterized. We have used the reversible carbon tetrachloride‐induced model of hepatocyte injury and liver fibrosis to show that in two models of genetic 11βHSD1 deficiency (global, *Hsd11b1* ^–/–^, and hepatic myofibroblast‐specific, *Hsd11b1* ^fl/fl^/Pdgfrb‐cre) 11βHSD1 pharmacological inhibition *in vivo* exacerbates hepatic myofibroblast activation and liver fibrosis. In contrast, liver injury and fibrosis in hepatocyte‐specific *Hsd11b1* ^fl/fl^/albumin‐cre mice did not differ from that of controls, ruling out 11βHSD1 deficiency in hepatocytes as the cause of the increased fibrosis. In primary HSC culture, glucocorticoids inhibited expression of the key profibrotic genes *Acta2* and *Col1α1*, an effect attenuated by the 11βHSD1 inhibitor [4‐(2‐chlorophenyl‐4‐fluoro‐1‐piperidinyl][5‐(1H‐pyrazol‐4‐yl)‐3‐thienyl]‐methanone. HSCs from *Hsd11b1* ^–/–^ and *Hsd11b1* ^fl/fl^/Pdgfrb‐cre mice expressed higher levels of *Acta2* and *Col1α1* and were correspondingly more potently activated. *In vivo* [4‐(2‐chlorophenyl‐4‐fluoro‐1‐piperidinyl][5‐(1H‐pyrazol‐4‐yl)‐3‐thienyl]‐methanone administration prior to chemical injury recapitulated findings in *Hsd11b1* ^–/–^ mice, including greater fibrosis. *Conclusion:* 11βHSD1 deficiency enhances myofibroblast activation and promotes initial fibrosis following chemical liver injury; hence, the effects of 11βHSD1 inhibitors on liver injury and repair are likely to be context‐dependent and deserve careful scrutiny as these compounds are developed for chronic diseases including metabolic syndrome and dementia. (Hepatology 2018;67:2167‐2181).

**Abbreviations**
ALT: alanine aminotransferase
αSMA: alpha‐smooth muscle actin
AST: aspartate aminotransferase
11βHSD1: 11beta‐hydroxysteroid dehydrogenase‐1
CD: cluster of differentiation
GC: glucocorticoid
GKO: global knockout
HSC: hepatic stellate cell
LKO: liver (hepatocyte)–specific knockout
MCDD: methionine/choline‐deficient diet
MFB: myofibroblast
MFKD: MFB/HSC‐specific 11βHSD1 knockdown
NAFLD: nonalcoholic fatty liver disease
NASH: nonalcoholic steatohepatitis
PSR: picrosirius red
TAA: thioacetamide
UE2316: [4‐(2‐chlorophenyl‐4‐fluoro‐1‐piperidinyl][5‐(1H‐pyrazol‐4‐yl)‐3‐thienyl]‐methanone
UE group: group receiving UE2316
UE‐R group: group receiving UE2316 only during resolution

The prevalence of chronic liver disease is increasing globally. Regardless of the underlying cause—alcohol, metabolic disease, or nonalcoholic steatohepatitis (NASH)—hepatic damage results in fibrosis, a dynamic process characterized by accumulation of extracellular matrix.^1^ Activated hepatic stellate cells/myofibroblasts (HSCs/MFBs) are the major source of extracellular matrix in mouse liver fibrosis models,^1^, ^2^ while scar‐associated macrophages facilitate the spontaneous resolution of liver fibrosis.^3^ The severity of fibrosis, for example, in NASH patients, is correlated with adverse clinical outcomes.^4^, ^5^ Currently, there is no effective regime to limit fibrosis without adversely affecting repair^4^; therefore, novel disease‐modifying antifibrotic therapies are needed.

Glucocorticoids (GCs) have wide‐ranging actions that modulate many of the pathological processes that occur during tissue injury and repair and contribute to liver fibrosis.^6^ Tissue GC levels are regulated by the intracellular enzyme 11beta‐hydroxysteroid dehydrogenase‐1 (11βHSD1), which converts inactive cortisone into active cortisol in humans (or 11dehydrocorticosterone into corticosterone in mice) and is highly abundant in liver.^7^ 11βHSD1 influences hepatic lipid accumulation, with transgenic 11βHSD1 overexpression in liver leading to hepatic steatosis and dyslipidemia and 11βHSD1 deficiency protecting from hepatic steatosis on a high‐fat diet.^8^, ^9^ However, little is known of the role of 11βHSD1 in liver fibrosis. One observational study showed no association between liver 11βHSD1 expression and the pathology of fatty liver or NASH in humans.^10^ In contrast, another study showed that in early stages of nonalcoholic fatty liver disease (NAFLD), with steatosis alone, hepatic 11βHSD1 activity is reduced, whereas progression to NASH was associated with increased 11βHSD1 levels.^11^

Importantly, 11βHSD1 inhibitors have been developed and shown to be moderately efficacious in phase 2 clinical trials in patients with type 2 diabetes.^12^ Moreover, a recent study showed that administration of the 11βHSD1 inhibitor RO5093151 in NAFLD patients reduced liver lipid content.^13^ Given the potential use of 11βHSD1 inhibitors as a therapy in patients either at risk of NAFLD or with established hepatic steatosis, it is imperative to understand the influence of 11βHSD1 on liver fibrosis.

In this study, we sought to define the direct effects of limiting liver GC availability on hepatic fibrosis, independent of metabolic functions. We therefore used global, hepatocyte‐specific, and HSC/MFB‐specific 11βHSD1–deficient mice and a specific small molecule 11βHSD1 inhibitor to study the functional role of 11βHSD1 in murine models of toxin‐induced liver fibrosis. We demonstrate that 11βHSD1 deficiency or inhibition promotes MFB activation and liver fibrogenesis in the CCl_4_ model.

## Materials and Methods

### MOUSE LIVER FIBROSIS MODELS

All experiments involving animals were approved by The University of Edinburgh Animal Welfare and Ethical Review Body and by the United Kingdom Home Office. Experiments used adult male (14 weeks of age) mice with global knockout (*Hsd11b1^−/−^*; GKO).^14^ GKO mice have been backcrossed for over 10 generations on a C57Bl/6J genetic background, and C57Bl/6J mice were used as controls.^15^, ^16^, ^17^, ^18^ GKO mice were maintained in parallel with the control C57Bl/6J mice; both lines were bred and maintained within our biomedical research facility, housed under standard conditions. To avoid interanimal variability that would be introduced due to differences in the stage of estrus in female mice, only male mice were used. Male mice (12 weeks of age) in which deletion was targeted to hepatocytes (liver‐specific knockout [LKO]) were generated by crossing *Albumin‐Cre^Tg^* ^/+^ mice (B6.Cg‐Tg[Alb‐cre]21Mgn/J; Jackson Laboratories) with mice homozygous for a “floxed” allele of *Hsd11b1* (*Hsd11b1^fl//fl^*) in which exon 3 is flanked by *LoxP* sites (generated by Artemis Pharmaceuticals directly onto a C57BL/6 background and designated *Hsd11b1^tm1Arte^*, MGI 5784734). Cre‐mediated excision of exon 3 generates a null allele.^19^ *Cre‐Hsd11b1^fl//fl^* littermates served as controls for LKO mice. To target deletion specifically at MFBs/HSCs (MFB/HSC‐specific 11βHSD1 knockdown [MFKD]), *Hsd11b1^fl//fl^* mice were crossed with *Pdgfrb‐Cre^Tg/+^* mice.^20^ *Cre‐Hsd11b1^fl//fl^* littermates served as controls for MFKD mice.

### CCl_4_ MODEL

Mouse chronic liver fibrosis was induced by intraperitoneal injection of 25% CCl_4_/g twice weekly for 12 weeks. Male GKO or LKO mice and their control littermates were culled at 24 hours (peak fibrosis), 72 hours, 1 week, and 1 month (scar resolution phases) after the last injection, as previously validated.^3^ MFKD male mice were culled 24 hours after the last CCl_4_ injection to evaluate the role of 11βHSD1 deficiency at the peak fibrotic response. For acute injury, a single dose of 25% CCl_4_/g intraperitoneally was given in GKO or control mice, and livers and plasma were collected after 24 hours.

In male C57Bl/6J mice, pharmacological 11βHSD1 inhibition was achieved by mixing a chow diet with 0.15% [4‐(2‐chlorophenyl‐4‐fluoro‐1‐piperidinyl][5‐(1H‐pyrazol‐4‐yl)‐3‐thienyl]‐methanone (UE2316). Groups of C57Bl6/J mice were given either a chow diet or a diet containing UE2316 (UE group) *ad libitum* 21 either throughout 12 weeks of CCl_4_ administration and until sacrifice or only from 48 hours after the last CCl_4_ injection until sacrifice, i.e., during resolution (UE‐R group). Mice were sacrificed at 6 hours, 24 hours, 72 hours, and 8 days after the last CCl_4_ injection.

### ALTERNATIVE LIVER PATHOLOGY MODELS

An alternative model of liver fibrosis was induced in C57Bl/6J mice by administration of thioacetamide (TAA; 600 mg/L) in drinking water for 1 year. Livers were harvested 24 hours or 1 week after TAA termination. To induce steatosis and NASH in a model of NAFLD, GKO male mice and age‐matched C57Bl/6J mice aged 6‐10 months were fed commercially available diets (all from Dyets, Bethlehem, PA)—control diet (518574), choline‐deficient diet (518753), or methionine/choline‐deficient diet (MCDD, 518810)—for 2 weeks, as described.^22^

### ISOLATION OF NONPARENCHYMAL LIVER CELLS AND FLOW CYTOMETRY

Fresh liver was perfused with 5 mL of saline through the portal vein at 100‐300 mg, then digested with Roswell Park Memorial Institute 1640 medium with deoxyribonuclease I and collagenase IV at 37^o^C. After digestion, hepatocytes were pelleted and discarded by centrifugation at 50*g* for 5 minutes. Nonparenchymal cells were washed with Roswell Park Memorial Institute 1640 medium and pelleted by centrifugation at 350*g* for 15 minutes, then washed and blocked with 10% mouse serum for 30 minutes. Antibodies against cluster of differentiation 11b (CD11b; clone M1/70; Ebioscience, Hatfield, UK), Ly‐6C (clone HK1.4; Ebioscience), CD45.2 (clone 104; Ebioscience), F4/80 (dilution 1:50; clone BM8; Invitrogen), and Ly‐6G (clone 1A8; Biolegend, San Diego, CA) were added for 30 minutes in a dilution of 1:100 unless otherwise specified. Cell viability was assessed with Fixable Viability Dye eFluor780 (Ebioscience) according to the manufacturer's protocols. Cells were formalin‐fixed and analyzed by flow cytometry using a BD LSR Fortessa II (BD Bioscience, Oxford, UK).

### IMMUNOFLUORESCENCE AND IMMUNOCHEMISTRY

For immunohistochemistry, livers were fixed with formalin for 16 hours directly after harvest. Sections, 4 μm, were dewaxed and rehydrated, followed by antigen retrieval in boiling sodium citrate. Avidin and biotin were blocked according to the manufacturer's protocol (Vector, Peterborough, UK). Protein block (Dako, Cambridge, UK) or serum was added, and sections were then incubated with alpha‐smooth muscle actin (αSMA; Sigma‐Aldrich, Dorset, UK), collagen I, or GR1 (Ly6G) (Cambridge Bioscience, Cambridge, UK), F4/80 (Abcam, Cambridge, UK), or reelin (Abcam) antibodies at 4^o^C overnight and subsequent secondary antibody for 30 minutes. After ABC vector incubation, sections were incubated with 3,3'‐diaminobenzidine for 10 minutes and counterstained with hematoxylin. Picrosirius red (PSR) staining was performed as described.^3^, ^23^ Thirty to 40 high‐power fields (magnification ×80) per section were randomly selected for each slide by an assessor blind to genotype. Images were analyzed in Photoshop CS3 for positively stained pixels and normalized to the total number of pixels. In the period acid–Schiff stain, the necrotic cell area was quantified in a blinded manner using the Image J Trainable Weka Segmentation tool.

### LIVER HISTOPATHOLOGY

Blinded hematoxylin and eosin–stained sections from the acute single‐dose CCl_4_ and the chronic CCl_4_ injury models (at 24 hours peak fibrosis) were evaluated by a pathologist using two separate ordinal scales. Inflammation was scored using the inflammation component of the NAFLD activity scoring system for NASH^24^ (0, no inflammation; 1‐<2, necroinflammatory foci/×20 field; 2‐3, necroinflammatory foci/×20 field; 3‐>4, necroinflammatory foci/×20 field). Hepatocellular necrosis was scored using a scale based on the descriptors of severity used in the reporting of acute lobular hepatitis in human biopsies (0, none; 1, single‐cell necrosis; 2, confluent necrosis; 3, zonal necrosis; 4, panacinar necrosis). The data are ordered categorically and, therefore, are presented as dot plots.

### PRIMARY CULTURE OF MOUSE HSCs

Livers from GKO, C57BL/6J control, MFKD, and control littermate mice were perfused with 5 mL of Hank's balanced salt solution, excised, cut into 2 × 2 mm^2^ cubes, and digested in Hank's balanced salt solution medium supplemented with deoxyribonuclease I, collagenase IV, and pronase. Released cells were purified through a 70‐μm strainer and separated by density gradient centrifugation in sequential concentrations of OptiPrep (Sigma‐Aldrich). Cells, 1 × 10^6^/well, were plated and cultured in Dulbecco's modified Eagle's medium with 16% fetal bovine serum and 1% penicillin/streptomycin. HSCs were left to spontaneously activate, then collected after 2, 5, 8, or 11 days in culture for mRNA and protein analysis. Pharmacological 11βHSD1 inhibition was performed in primary cultures of C57Bl/6J HSCs. HSCs were treated with vehicle, 500 nM corticosterone, 500 nM 11‐dehydrocorticosterone, and/or 10 μM UE2316. HSC medium was replaced daily, and all treatments were added daily with the medium change, for the duration of the experiment. Following 8 days of treatment, HSCs were collected for mRNA and protein analysis.

### OTHER STANDARD METHODS

Immunoblotting, RNA extraction, real‐time PCR, 11βHSD1 activity assay, and *in situ* hybridization have been described^25^, ^26^, ^27^; and detailed methods can be found in the http://onlinelibrary.wiley.com/doi/10.1002/hep.29734/suppinfo.

### STATISTICAL ANALYSIS

All data are expressed as mean ± SEM. Statistical analysis was performed using GraphPad prism or Statistica 7. Two‐way analysis of variance was used to test for the interaction of genotype/treatment analysis. Two‐tailed unpaired Student *t* test or one‐way analysis of variance was used for the comparison of two (i.e., control versus knockout mice) or three (i.e., control, chow diet, MCDD comparisons) groups, respectively.

## Results

### 11βHSD1 EXPRESSION IS REDUCED DURING HEPATIC INJURY

**Figure 1:**
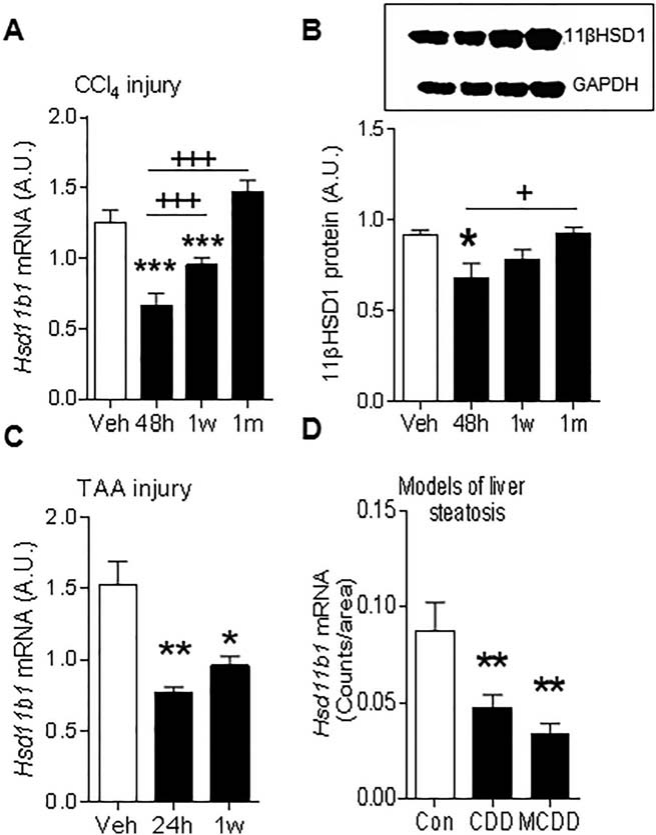
Reduced 11βHSD1 expression in mouse models of liver fibrosis. Hepatic 11βHSD1 mRNA levels measured by real‐time PCR (A) and protein levels (B), with upper panel showing representative immunoblot for 11βHSD1 and glyceraldehyde 3‐phosphate dehydrogenase and lower panel quantification graph, during peak (48 hours) and resolution (1 week, 1 month) phases in the reversible CCl_4_ (A,B) and TAA (C) liver fibrosis models, n = 6/group. (D) Hepatic *Hsd11b1* mRNA levels in control diet, choline‐deficient diet, and MCDD NASH models, assessed by *in situ* mRNA hybridization (quantification graph showing average grain counts per area), n = 6/group. ^*^ *P* < 0.05, ^**^ *P* < 0.01, ^***^ *P* < 0.001 compared to the vehicle group (A‐C); ^+^ *P* < 0.05, ^+++^ *P* < 0.001 compared to the 48‐hour injury time point. Abbreviations: A.U., arbitrary unit; GAPDH, glyceraldehyde 3‐phosphate dehydrogenase.

In the CCl_4_ murine model of liver fibrosis, whole‐liver 11βHSD1 mRNA (Fig. 1A) and protein (Fig. 1B) levels were reduced during injury. This injury‐associated reduction in whole‐liver 11βHSD1 mRNA was recapitulated in both the TAA (Fig. 1C) model of liver fibrosis and in the choline‐deficient diet and MCDD models of steatosis and NASH (Fig. 1D). 11βHSD1 activity levels were not significantly altered during peak CCl_4_‐induced fibrosis (http://onlinelibrary.wiley.com/doi/10.1002/hep.29734/suppinfo). During the scar resolution phase following CCl_4_ injury, 11βHSD1 mRNA and protein levels returned to levels seen in uninjured (vehicle‐treated) mice (Fig. 1A,B).

### 11βHSD1 IS EXPRESSED IN HSCs, ATTENUATING THEIR ACTIVATION *IN VITRO*

**Figure 2:**
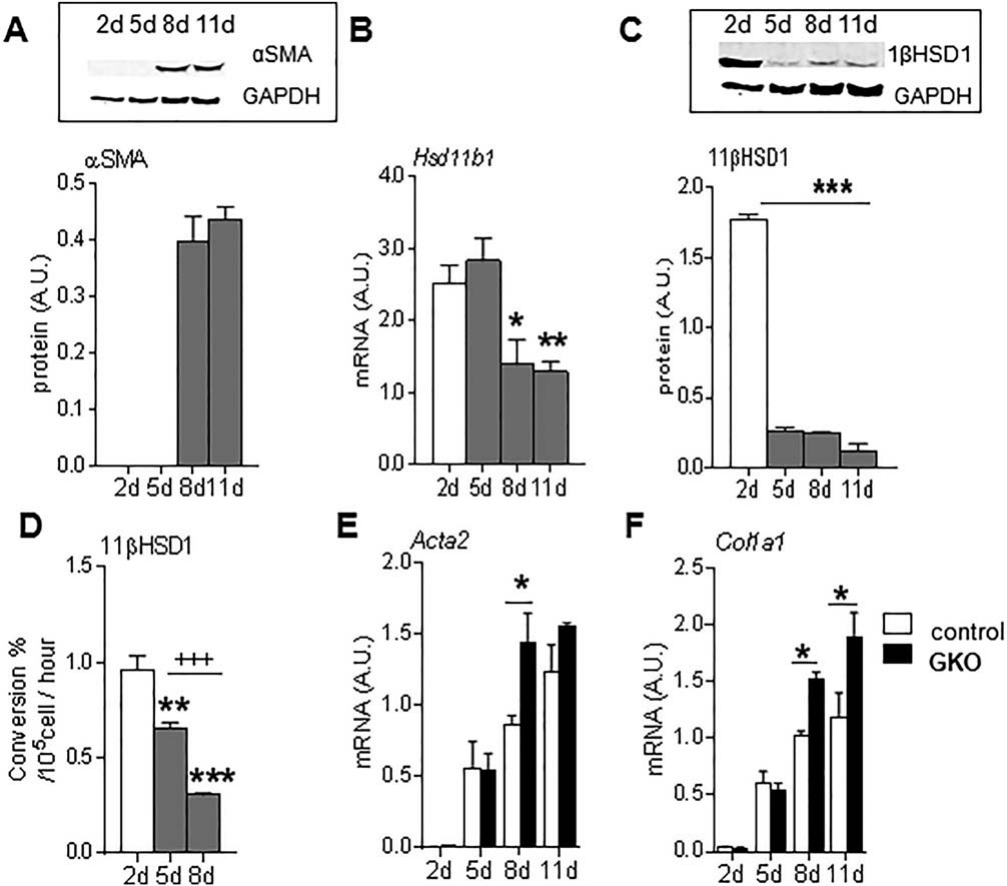
Reduced 11βHSD1 expression during HSC activation *in vitro*. Expression levels of 11βHSD1 and αSMA during *ex vivo* wild‐type mouse HSC activation *in vitro* (n = 3/group). (A) αSMA protein, (B) *Hsd11b1* mRNA, (C) 11βHSD1 protein, and (D) 11βHSD1 enzymatic activity (percent conversion of 11‐dehydrocorticosterone to corticosterone) were measured during 2‐11 days postactivation. Comparison of *Acta2* (E) and *Col1a1* (F) mRNA levels in wild‐type (white bars) and GKO (black bars) HSCs during 2‐11 days postactivation. ^*^ *P* < 0.05, ^**^ *P* < 0.01, ^***^ *P* < 0.001 compared to basal day 2 (A‐D) or between genotypes within a time point (E,F); ^+++^ *P* < 0.001 comparisons between days 5 and 8 (D). Abbreviations: A.U., arbitrary unit; GAPDH, glyceraldehyde 3‐phosphate dehydrogenase.

Although 11βHSD1 is highly expressed in hepatocytes, the key cells orchestrating fibrogenesis in parenchymal liver injury models are the HSCs. HSCs are “spontaneously” activated in culture such that αSMA (a global marker of spontaneous HSC activation *in vitro*) protein levels are maximal at day 8 (Fig. 2A). Significant 11βHSD1 expression was detectable in primary murine HSCs, with a striking reduction observed in 11βHSD1 mRNA (Fig. 2B), protein (Fig. 2C), and activity (Fig. 2D) following spontaneous HSC activation (day 8) *in vitro*. Similarly, 11βHSD1 gene expression was present in the human LX‐2^28^ HSC cell line and was significantly reduced in response to activation with transforming growth factor beta, a classic profibrogenic stimulus (http://onlinelibrary.wiley.com/doi/10.1002/hep.29734/suppinfo).

To investigate the functional role of 11βHSD1 in HSC activation, primary HSCs were isolated from GKO (11βHSD1‐deficient) mice and showed significantly higher *Acta2* (Fig. 2E) and *Col1a1* (Fig. 2F) mRNA levels at day 8 compared to control HSCs.

**Figure 3:**
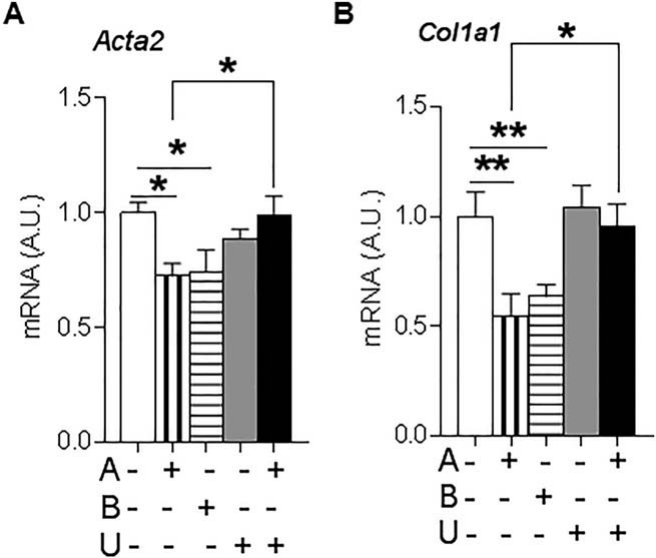
GC inhibition of profibrotic gene expression is amplified by 11βHSD1 in murine HSCs *in vitro*. mRNA levels of *Acta2* (A) and *Col1α1* (B) in C57Bl6/J HSCs in primary culture for 8 days treated with combinations of vehicle (white bars), 500 nM 11‐dehydrocorticosterone (compound A, vertical striped bars), 500 nM corticosterone (compound B, horizontal striped bars), 10 μM UE2316 (compound U, gray bars), and the combination of A+B (black bars). Levels of mRNAs of interest are expressed relative to 18S RNA. Experiments were conducted in triplicate: ^*^ *P* < 0.05, ^**^ *P* < 0.01. Abbreviation: A.U., arbitrary unit.

Consistent with this effect of 11βHSD1 being mediated by amplification of GCs, incubation of wild‐type murine HSCs with either corticosterone or 11‐dehydrocorticosterone, the inert substrate for 11βHSD1, reduced *Acta2* (Fig. 3A) and *Col1a1* (Fig. 3B) mRNA levels. The effects of 11‐dehydrocorticosterone were abrogated by coadministration of the specific 11βHSD1 inhibitor UE2316 (Fig. 3A,B).

### GLOBAL 11βHSD1‐DEFICIENT MICE SHOW INCREASED HEPATIC MFB ACTIVATION FOLLOWING CCl_4_ INJURY

**Figure 4:**
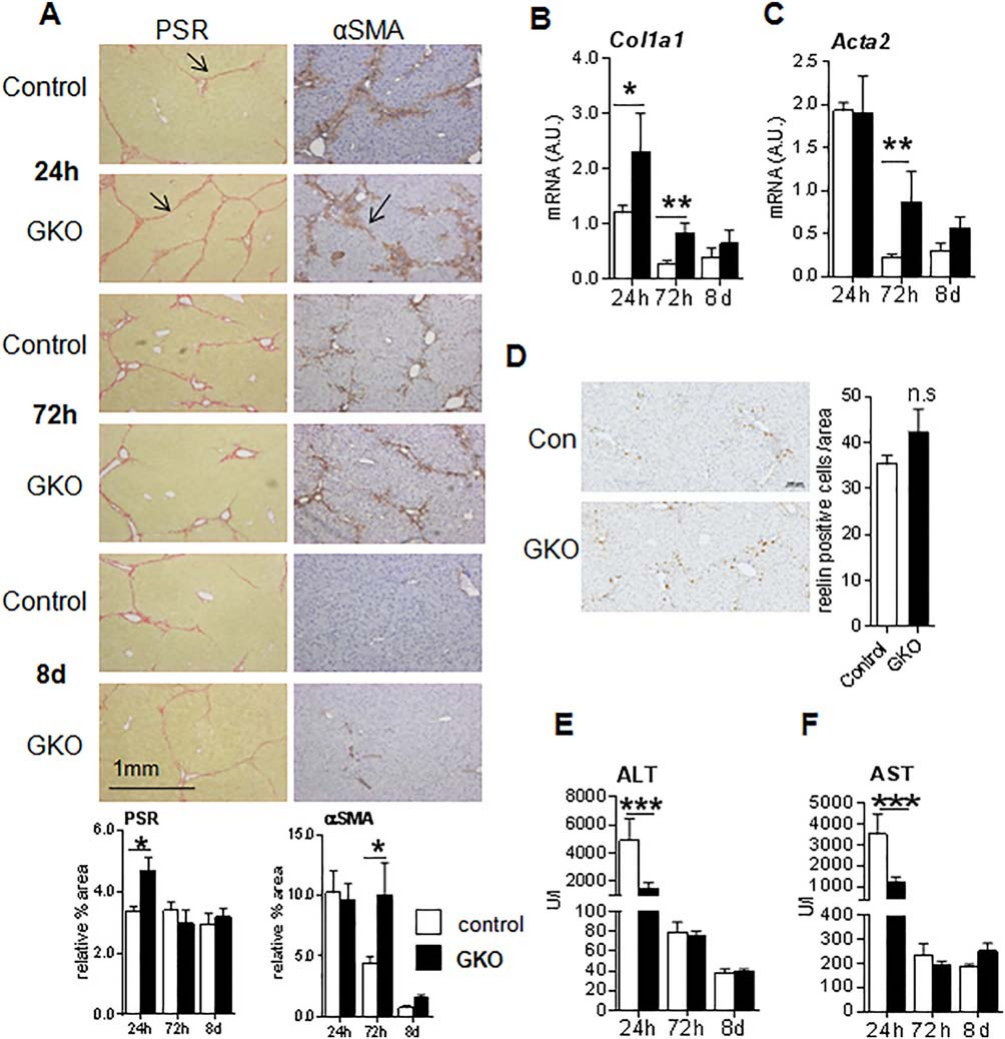
Increased fibrotic response in livers of GKO mice after CCl_4_ administration. GKO (black bars) and wild‐type (white bars) mice were administered twice weekly intraperitoneal injections of CCl_4_ for 12 weeks. The 24‐hour time point represents the peak injury, and 72 hours to 8 days is the scar resolution phase. (A) Representative images of total collagen (PSR), and αSMA staining during injury (24 hours) and resolution (72 hours and 8 days) phases, with arrowheads pointing to intense collagen staining and αSMA‐positive cells in control and GKO mice. Quantification of staining by PSR and αSMA is shown below the corresponding panel of representative images. Pixels of 30‐40 continuous fields (×80) from each section were randomly selected and measured. Hepatic levels of *Col1a1* (B) and *Acta2* mRNA (C) were measured by quantitative PCR and normalized for 18S. (D) Representative images (scale bar, 100 μm) and quantification graph of reelin immunohistochemistry (at 72 hours) to identify HSCs. Plasma levels of ALT (E) and AST (F) during injury and resolution phases. n = 5‐6 in each group; ^*^ *P* < 0.05, ^**^ *P* < 0.01, ^***^ *P* < 0.001 between genotypes within time points. Abbreviation: A.U., arbitrary unit.

To investigate whether reducing 11βHSD1 (in GKO mice) *in vivo* enhances MFB/HSC activation and delays the resolution of scarring, we used the reversible CCl_4_ liver fibrosis model because the times of peak injury and maximal scar resolution have been well defined; time points were chosen to reflect the injury and resolution phases because collagen deposition is higher at 24 hours and resolves after 72 hours.^3^ After 12 weeks of CCl_4_ treatment, GKO mice showed significantly higher PSR staining (Fig. 4A) and collagen I deposition (http://onlinelibrary.wiley.com/doi/10.1002/hep.29734/suppinfo) at peak (24 hours) fibrosis. Increased fibrogenesis in the absence of 11βHSD1 was supported by elevated liver mRNA levels of *Col1a1* (Fig. 4B). In keeping with enhanced MFB activation, western blot analysis showed that αSMA protein levels were higher at 24 hours and stayed elevated at 8 days in GKO mice (http://onlinelibrary.wiley.com/doi/10.1002/hep.29734/suppinfo). Similarly, histological quantification of αSMA immunostaining showed that GKO mice retained higher αSMA during the resolution phase (72 hours; Fig. 4A), although no histological difference was detected at peak fibrosis. In addition, *Acta2* (αSMA) gene expression remained higher in GKO mice during resolution (72 hours; Fig. 4C). To identify whether the observed alteration in MFB phenotype was specific to HSCs, we stained for reelin (Fig. 4D), a known marker of HSCs.^29^ We did not detect any significant difference in the number of reelin‐positive cells at 72 hours, the critical point at which markers of fibrosis were elevated in GKO mice. Hence, no definitive conclusion could be drawn on the origins of MFBs in this model.

### GKO MICE SHOW SIMILAR HEPATOCELLULAR DAMAGE AFTER CHRONIC OR ACUTE LIVER INJURY

To assess whether the exaggerated profibrotic response in GKO mice following chronic CCl_4_ was due to more severe hepatocyte injury, we performed a detailed histopathological evaluation. This showed similar NAFLD activity scores for inflammation, hepatocellular necrosis, and total injury in GKO and control mice (http://onlinelibrary.wiley.com/doi/10.1002/hep.29734/suppinfo). In fact, plasma alanine aminotransferase (ALT) and aspartate aminotransferase (AST) levels were lower in GKO mice compared to control injured mice during peak fibrosis (Fig. 4E,F). Plasma albumin levels were comparable between genotypes at the peak injury (24 hours) time point (control versus GKO, 25.40 ± 2.12 U/L versus 22.80 ± 0.66 U/L) and remained at similar levels throughout the resolution phases (data not shown).

To further evaluate the effects of global 11βHSD1 deficiency on hepatocellular injury, a single dose of CCl_4_ was administered to induce acute liver injury. As in the chronic CCl_4_ model, the GKO mice showed significantly lower plasma ALT, AST, and alkaline phosphatase but similar albumin levels (http://onlinelibrary.wiley.com/doi/10.1002/hep.29734/suppinfo). The single dose confirmed similar histopathological scores between genotypes (http://onlinelibrary.wiley.com/doi/10.1002/hep.29734/suppinfo). Quantification of hepatocyte necrotic cell area using period acid–Schiff staining showed similar degrees of hepatocellular death in GKO and control mice (http://onlinelibrary.wiley.com/doi/10.1002/hep.29734/suppinfo).

### GKO MICE SHOW SIMILAR HEPATIC MACROPHAGE OR NEUTROPHIL NUMBERS TO CONTROL MICE IN CCl_4_ INJURY

**Figure 5:**
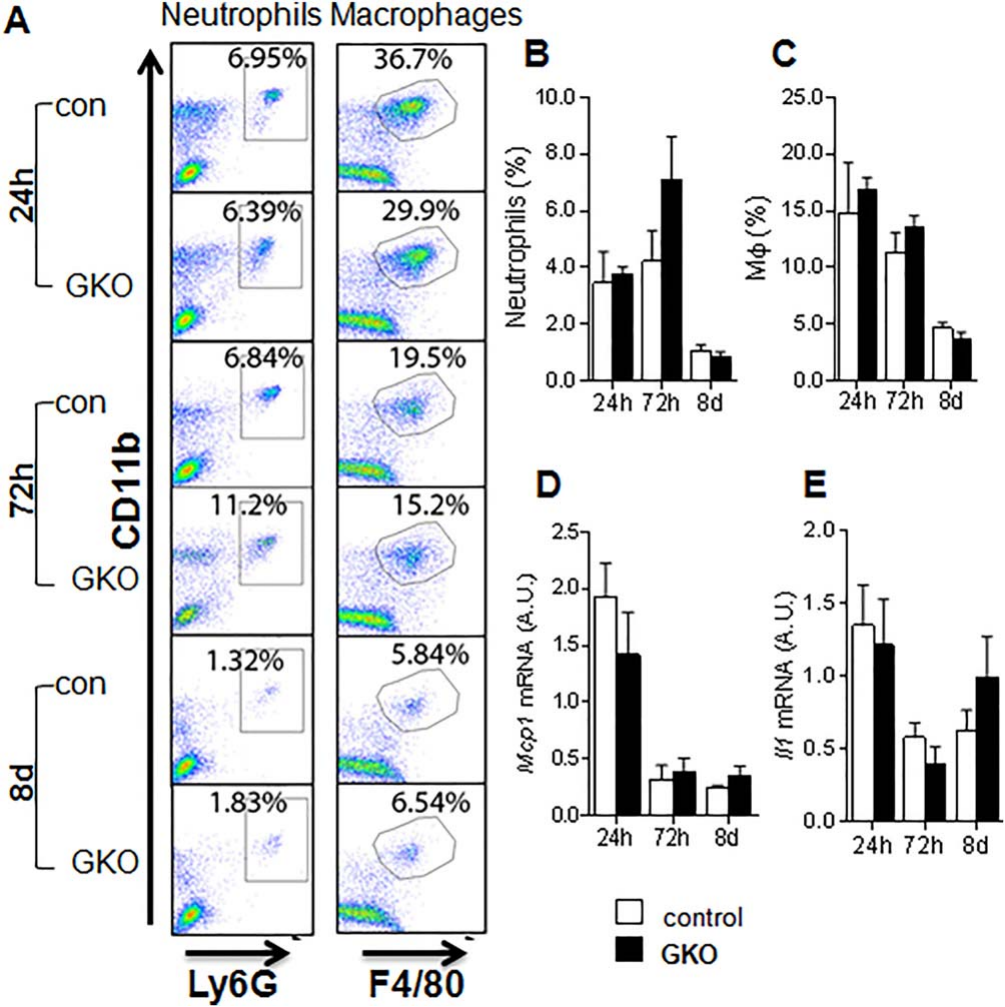
Similar inflammatory responses to control mice in GKO following CCl_4_ liver injury. (A) A panel of representative flow‐cytometric images gating for Ly6G^+^CD11b^+^ cells (neutrophils) and F4/80^+^CD11b^+^ cells (macrophages) with quantification graphs for inflammatory cells as percentage of neutrophils (B) and macrophages (C) among all nonparenchymal cells in the liver. Hepatic levels of mRNAs encoding monocyte chemoattractant protein‐1 (D) and interleukin‐1beta (E) quantified by quantitative PCR and normalized for 18S in control (white bars) and GKO (black bars) mice. There were no differences between groups (n = 6/group). Abbreviations: A.U., arbitrary unit; Il1, interleukin 1; Mcp1, monocyte chemoattractant protein‐1.

Apart from HSCs, macrophages have been implicated in both promoting fibrosis and facilitating resolution; therefore, we further assessed if global 11βHSD1 deletion affects hepatic macrophages and neutrophil numbers during injury and resolution phases. Neutrophil numbers were similar between GKO and control livers at all time points (Fig. 5A,B). Macrophage numbers (Fig. 5A‐C) and cytokine mRNA levels of *Mcp1* and *Il1* (Fig. 5D,E) were also similar in GKO and control mice. Taken together these data suggest that enhanced profibrotic response in GKO mice is not due to exacerbated inflammation or macrophage‐induced fibrosis but is more likely to reflect a direct effect on HSC activation pathways.

### MFB/HSC 11βHSD1 KNOCKDOWN ENHANCES FIBROSIS MARKERS IN RESPONSE TO CHRONIC CCl_4_

**Figure 6:**
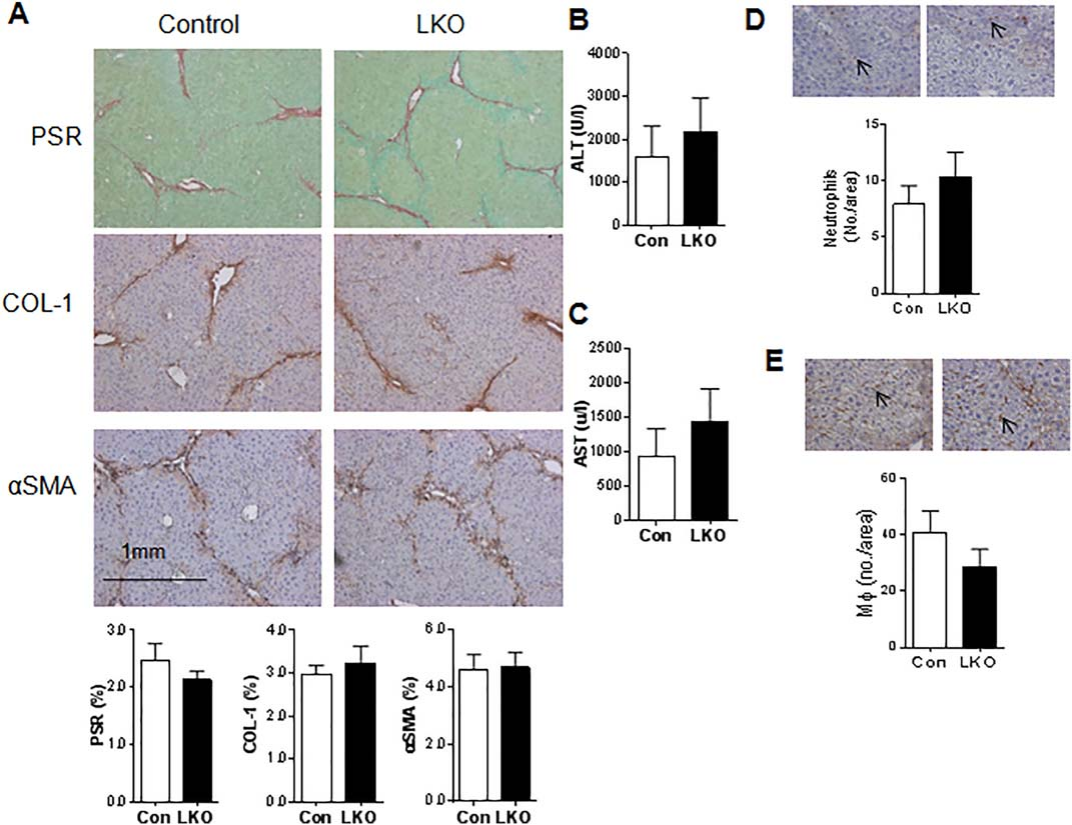
Mice with hepatocyte‐specific 11βHSD1 deficiency (LKO) and their littermate controls show similar hepatic responses to CCl_4_ injury. (A) Representative images of total collagen (PSR), Col1, and αSMA staining at 24 hours peak injury in control and LKO mice with quantification graphs at the bottom of each representative image panel. Plasma levels of ALT (B) and AST (C) in control (*Hsd11b1* ^fl/fl^; white bars) and LKO (black bars) mice during injury. There were no differences between groups (n = 6/group). Control (white bars) and LKO (black bars) liver sections were stained for neutrophils GR‐1 (Ly6G; D) and macrophages (F4/80; E). For each section 20‐30 consecutive fields with magnification ×400 were quantified. Cells in each field were counted by an investigator, who was blinded for the genotype and treatment of the section. The average cell number per field was calculated. There were no differences between groups (n = 5‐6/group).

To distinguish between possible contributions of 11βHSD1 deficiency in MFBs and hepatocytes to the profibrotic phenotype, we generated two independent mouse models targeting 11βHSD1 in either hepatocytes or MFBs/HSCs. Mice with hepatocyte‐specific deficiency in 11βHSD1 (LKO) showed almost complete (94%‐100% knockdown) loss of 11βHSD1 (http://onlinelibrary.wiley.com/doi/10.1002/hep.29734/suppinfo). At 24 hours after chronic CCl_4_ the LKO mice showed identical hepatic profibrotic responses histologically (Fig. 6A), similar liver function test changes (Fig. 6B,C), and identical macrophage or neutrophil numbers (Fig. 6D,E) to control littermates. Thus, deletion of 11βHSD1 in hepatocytes does not mimic the changes in liver fibrosis in GKO mice.

**Figure 7:**
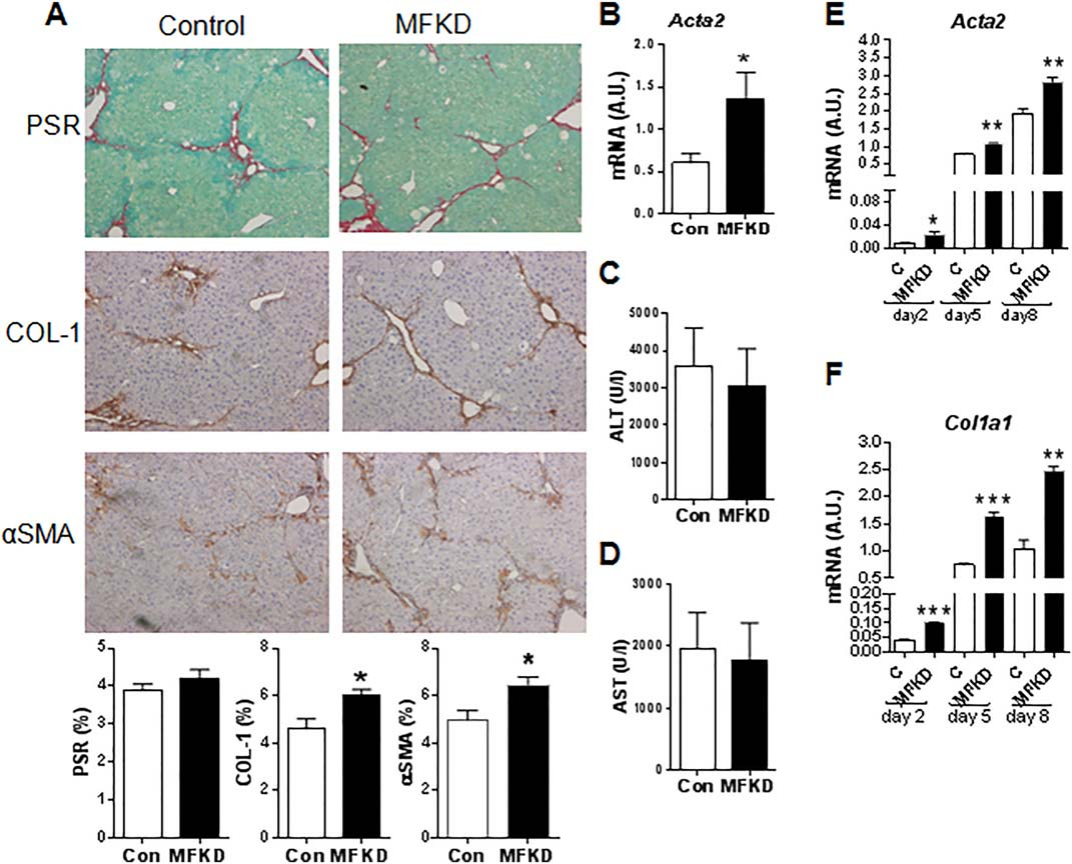
Knockdown of 11βHSD1 specifically in HSC/MF populations (MFKD) enhances hepatic MFB activation and fibrotic response in CCl_4_ injury. Male mice (10‐12 weeks old) were administered CCl_4_ intraperitoneally for 12 weeks to induce liver injury (n = 6/group). (A) Representative images of total collagen (PSR), Col1, and αSMA staining at 24‐hour peak injury in control and MFKD mice with quantification graphs at the bottom of each representative image panel. (B) Hepatic *Acta2* mRNA levels were measured by quantitative PCR and normalized for 18S. Plasma levels of ALT (C) and AST (D) in control (*Hsd11b1* ^fl/fl^; white bars) and MFKD (black bars) mice during injury. HSCs were isolated from 8‐week‐old mice. *Acta2* (E) and *Col1a1* (F) mRNA levels were measured at 2, 5, and 8 days post *ex vivo* HSC activation in MFKD (black bars) and control littermates (white bars) (n = 3/group). ^*^ *P* < 0.05, ^**^ *P* < 0.01, ^***^ *P* < 0.001 comparisons between genotypes. Abbreviation: A.U., arbitrary unit.

To assess the specific role of 11βHSD1 in the regulation of MFBs/HSCs in the CCl_4_ model, we used MFKD mice. HSCs isolated from MFKD mice demonstrate a 50%‐60% reduction in 11βHSD1 expression *in vitro* (http://onlinelibrary.wiley.com/doi/10.1002/hep.29734/suppinfo) compared to littermate controls. Following chronic CCl_4_ administration, MFKD mice showed increased COL1 and αSMA immunostaining (Fig. 7A) and significantly higher *Acta2* mRNA (Fig. 7B) levels at 24 hours post–CCl_4_ liver injury. No significant difference was detected in PSR staining (Fig. 7A) or plasma ALT/AST levels (Fig. 7C,D) between genotypes. Enhanced MFB activation in MFKD mice was also confirmed in *ex vivo* primary HSCs from MFKD mice that showed significantly higher *Acta2* and *Col1a1* levels at days 2, 5, and 8 in culture (Fig. 7E,F).

### PHARMACOLOGICAL 11βHSD1 INHIBITION INCREASES MFB ACTIVATION AND EARLY FIBROSIS BUT ENHANCES SCAR RESOLUTION

**Figure 8:**
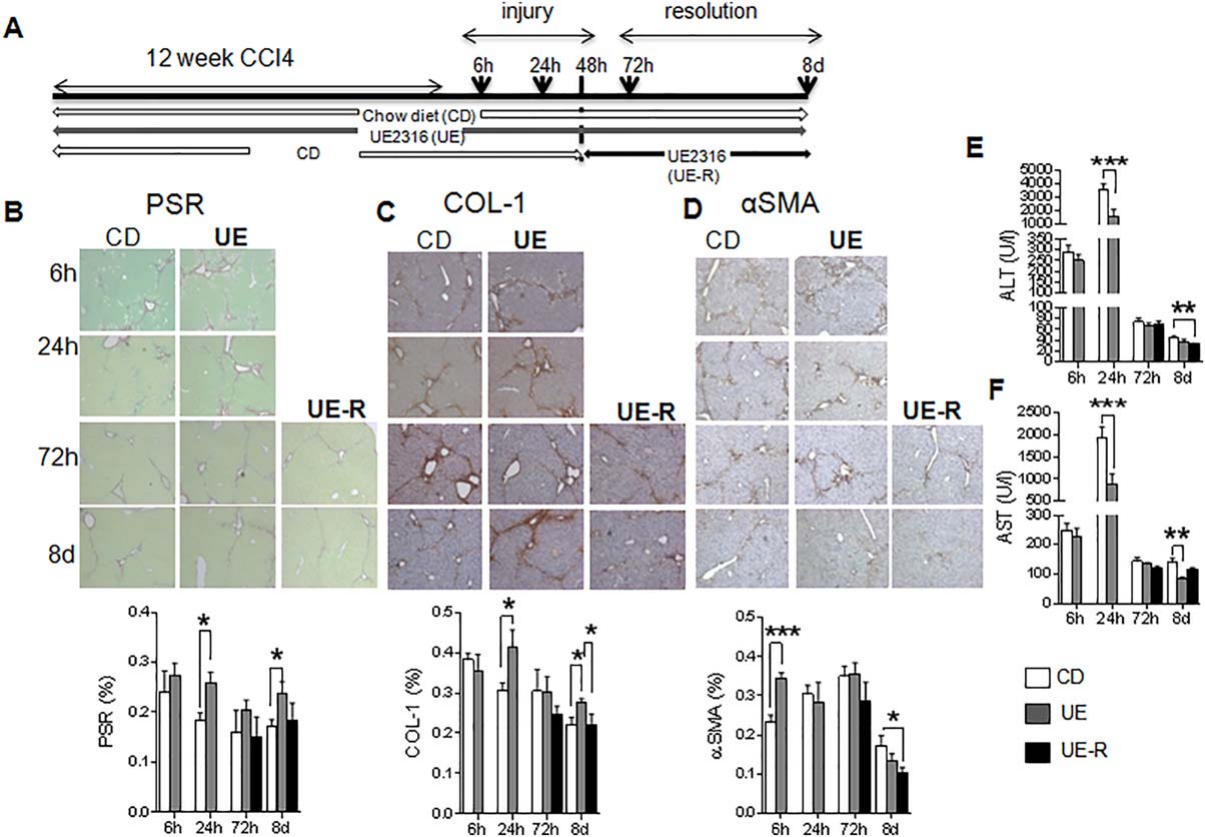
Inhibition of 11βHSD1 in mice enhances the early liver fibrotic response to CCl_4_ but, when restricted to the repair phase, improves resolution of scarring. (A) Schematic view of the experimental design. Mice were administered twice weekly intraperitoneal injections of CCl_4_ for 12 weeks and divided into three groups: a chow diet (white) group that was kept on vehicle diet for the duration of the experiment, a UE group (gray) that was kept on a diet containing the 11βHSD1 inhibitor UE2316 for the whole duration of the experiment, and a UE‐R group (black) that started on a vehicle chow diet and switched to the UE diet 48 hours after the last CCL_4_ injection. In this experiment a very early 6‐hour time point was included to capture the earliest injury response. Representative images and quantification graphs of PSR (B), Col1 (C), and αSMA (D) staining were quantified in 30‐40 continuous fields (×80) from each section of each group. Plasma levels of ALT (E) and AST (F) in mice treated with vehicle (white bars), UE (gray bars), or UE‐R (black bars). n = 5‐6 in each group; ^*^ *P* < 0.05, ^**^ *P* < 0.01, ^***^ *P* < 0.001 tested by two‐way analysis of variance. Abbreviation: CD, chow diet.

To address the clinical relevance of our findings for 11βHSD1 inhibitor therapies and specifically test the potential intervention times (prophylactic/injury/repair), we used the 11βHSD1 inhibitor UE2316 in the CCl_4_ liver fibrosis model (Fig. 8A). During CCl_4_ administration, UE2316‐treated animals had slightly lower body weights (http://onlinelibrary.wiley.com/doi/10.1002/hep.29734/suppinfo)^21^ but no difference in liver‐to‐body weight ratio (http://onlinelibrary.wiley.com/doi/10.1002/hep.29734/suppinfo). Overall, as observed in GKO mice, UE2316 treatment throughout the period of CCl_4_ administration exacerbated hepatic fibrosis measured 24 hours following termination of CCl_4_, as shown by PSR staining (Fig. 8B) and collagen 1 (Fig. 8C) immunohistochemistry. UE2316 treatment throughout CCl_4_ injury also inhibited later scar resolution, as shown by elevated PSR and collagen 1 up to 8 days post‐CCl_4_. The UE2316‐treated group had higher αSMA as early as 6 hours after the last CCl_4_ injection, but this normalized during later resolution (Fig. 8D). Furthermore, as seen in GKO mice, plasma ALT and AST levels were significantly lower at both peak injury (24 hours) and late resolution (day 8) in the UE group compared to vehicle (Fig. 8E,F).

In order to investigate the therapeutic potential of 11βHSD1 inhibition and/or dissect the effects on injury/repair phases, we administered UE2316 only during scar resolution, i.e., commencing 48 hours following the final CCl_4_ injection (Fig. 8A).There was no significant difference in total collagen or collagen 1 deposition in the UE‐R group (Fig. 8B,C) compared to vehicle control, but administering UE2316 during the resolution phase accelerated the reduction in the number of αSMA‐positive cells (Fig. 8D) and reduced plasma ALT levels (Fig. 8E) at day 8.

## Discussion

Collectively, these data from a comprehensive series of liver fibrosis studies using mice with either global or MFB‐restricted 11βHSD1 deficiency, a translational study using a small molecule 11βHSD1 inhibitor, and our *in vitro* work using primary HSCs demonstrate that attenuation of 11βHSD1 activity within the hepatic MFB population enhances a profibrogenic MFB phenotype and promotes liver fibrogenesis. The profibrotic response most likely occurs in HSCs and not in hepatocytes (LKO had no phenotype) and is mediated by suppression of GC action through 11βHSD1inhibition. It is also evident that in all the models of liver injury that we assessed, 11βHSD1 is suppressed during injury. Therefore, administration of systemic 11βHSD1 inhibitors, drugs which are currently under investigation in phase 2 clinical trials, to patient groups at risk of NAFLD or NASH may have unpredictable adverse effects on liver fibrosis progression and regression.

The profibrotic responses shown with GKO and pharmacological inhibition are not due to greater hepatocellular injury because histopathology and necrosis scores did not show marked differences in liver damage. Interestingly, biochemical markers of hepatocyte death (ALT and AST) were reduced in 11βHSD1 global deficiency/inhibition. The lower ALT/AST at peak fibrosis in our study is currently unexplained. However, given the role of 11βHSD1 in the modulation of anti‐inflammatory GCs, we speculate that this could relate to differential effects of reduced GC exposure (systemically versus locally). Indeed, administration of exogenous GC to patients has been shown to increase ALT and AST levels.^30^ Animal studies have also shown that GCs increase ALT activity^31^, ^32^; therefore, reduced GC availability with 11βHSD1 pharmacological inhibition/global genetic deficiency could explain the reduced ALT/AST. In the cell type (LKO, MFKD)–specific models, the ALT/AST levels were similar to littermate controls. Hence, ALT/AST levels may not always reflect the degree of hepatocellular damage, and histological confirmation of changes should be sought where possible. Note that the ALT and AST levels differed in the respective control strains, likely because of background stain and age differences; this does not change the interpretation of our data.

Hepatocyte‐specific deletion of 11βHSD1 did not show any apparent effect on liver fibrosis or hepatic injury in the CCl_4_ injury model, ruling out hepatocytes as a key locus for 11βHSD1 in this context. Similarly, in a fatty liver model, LKO mice failed to show significant metabolic abnormalities or a distinct phenotype from control littermates.^33^ It is possible that in global deficiency compensatory hypothalamic–pituitary–adrenal axis activation (due to increased GC clearance) or paracrine modulators could affect profibrotic responses. For example, global 11βHSD1 deficiency or inhibition, but not hepatocyte‐specific deficiency,^33^, ^34^ leads to moderate weight loss, which may modulate injury responses in the liver by altered leptin signaling.^35^ This suggests that 11βHSD1 deficiency, either in other organs or in other cells within the liver, is important to modifying the injury response.

Our data using the MFKD mouse model and *ex vivo* HSC data (from MFKD, GKO, UE2316) suggest that 11βHSD1 could be a key regulator of MFB/HSC function. Reduction of 11βHSD1 in MFB/HSC populations is permissive toward a profibrotic profile with increased collagen 1 (a GC target gene) and αSMA levels. The lack of a significant difference in PSR staining in the MFKD model could be due to residual levels (50%‐60% knockdown) of 11βHSD1 in MFBs/HSCs. The inhibitory effects of GCs on profibrotic genes have also been observed in other disease models where fibroblasts play a key role. For example, in transforming growth factor beta–stimulated human lung fibroblasts, GC treatment significantly reduced extracellular matrix–related genes.^36^ GCs also inhibit the proinflammatory cytokine‐induced proliferation of adult rat cardiac fibroblasts.^37^ In support of our data, dexamethasone treatment of HSCs resulted in a significant reduction of Tgf‐β‐Smad‐mediated signaling,^38^ the upstream regulator of matrix deposition by activated HSCs. Although the effects of MFKD, 11βHSD1 genetic deficiency, and pharmacological inhibition suggest direct activation of HSCs *in vitro* and the fact that HSCs are the major source of MFBs in hepatotoxic liver injury (CCl_4_),^39^, ^40^ it is possible that other hepatic MFB subpopulations could have contributed to the effects observed in our *in vivo* studies.

Hepatic inflammation is also closely linked to liver fibrosis. Liver macrophages are critical regulators of both fibrogenesis and scar resolution.^3^, ^41^ GCs are well known for their anti‐inflammatory properties, and 11βHSD1 is expressed in macrophages and other inflammatory cells.^42^ Effects of 11βHSD1 on inflammation are highly context‐dependent. For example, GKO mice show reduced inflammation in atheroma lesions and decreased monocyte chemoattractant protein 1 expression and macrophage numbers in adipose tissue^16^ after high‐fat feeding but enhanced responses in serum arthritis and chemically induced peritonitis and pleurisy^42^ as well as increased serum tumor necrosis factor‐alpha and interleukin‐6 levels when challenged with lipopolysaccharide^43^ and enhanced hepatic inflammation in an NAFLD diet‐inducing model.^44^ We suspected that the profibrotic profile seen in GKO and UE2316‐treated mice could be attributed to exaggerated inflammatory response to CCl_4_. However, we did not find any elevation of key inflammatory markers or of either hepatic macrophage or neutrophil numbers. Therefore, in our model it is unlikely that 11βHSD1 deficiency plays a regulatory role on inflammatory cell function.

We have not excluded other mechanisms potentially affecting fibrosis. In contrast with permissive effects on liver fibrosis in CCl_4_ injury, GKO mice showed protection from adipose tissue scarring after high‐fat feeding. This antifibrotic role is associated with enhanced angiogenesis and reduced hypoxia in the adipose tissue of GKO mice,^17^ consistent with their enhanced angiogenesis in models of ischemic or injured tissue.^15^, ^18^, ^45^ In chronic liver disease several studies show that angiogenesis is key in fibrosis progression, with exaggerated pathological angiogenesis leading to enhanced fibrosis.^46^, ^47^, ^48^, ^49^ Therefore, increased angiogenesis with reduced 11βHSD1 in the liver could contribute to the enhanced fibrosis we observed.

Our work provides insights into the translational importance of the use of 11βHSD1 inhibitors in liver injury. Administration of UE2316 prior to injury recapitulates the profibrotic phenotype seen in the GKO and MFKD genetic models. UE2316 restricted to the recovery phase, after chronic injury had been stopped, seemed to modestly reduce markers of MFB activation. This dichotomous function of 11βHSD1 during injury and resolution suggests context‐dependent treatment effects. Similar paradigms for other pathways are now becoming more evident in the literature. For example, a recent study using vascular endothelial growth factor–neutralizing antibodies showed two opposing effects: prevention of the development of fibrosis but also disruption of hepatic fibrosis resolution and tissue repair. During fibrosis resolution, vascular endothelial growth factor inhibition impaired liver sinusoidal permeability, which was associated with reduced monocyte infiltration of fibrotic liver and delayed tissue repair.^50^ GCs have known angiostatic mechanisms, and we have shown that 11βHSD1 inhibition enhances vascular endothelial growth factor expression, which might be necessary during extracellular matrix remodeling at the scar resolution phase.

Selective 11βHSD1 inhibitors have been tested in phase 2 trials of obese patients with type 2 diabetes, but the modest size of their effects on glycemic control have stalled their commercial progress and led to consideration of alternative/added indications, including NAFLD.^13^ From these preclinical data we cannot predict the consequences of 11βHSD1 inhibition for liver injury, and this requires more careful investigation. From a therapeutic point of view, it appears that administration of 11βHSD1 inhibitors prior to injury (as a prophylactic measure) might reduce transaminase levels but will perhaps be permissive toward fibrosis and delay collagen degradation. In contrast, if it were possible to administer 11βHSD1 inhibitors specifically during scar resolution, this may assist extracellular matrix remodeling.

Author names in bold designate shared co‐first authorship.

## Supporting information

Additional Supporting Information may be found at http://onlinelibrary.wiley.com/doi/10.1002/hep.29734/suppinfo.

Supporting Information 1

Supporting Information Figure Caption
