# Supplementary material for: 11Beta‐hydroxysteroid dehydrogenase‐1 deficiency or inhibition enhances hepatic myofibroblast activation in murine liver fibrosis
Source: Hepatology. 2018 Feb 22;67(6):2167–81. doi: 10.1002/hep.29734 (PMC6001805; doi:10.1002/hep.29734)

HEP16-2216 Supporting Methods

Immunoblotting. Livers and HSCs were homogenized in lysis buffer (0.15M NaCl, 0.05M HEPES, 1% Triton X-100, 10% Glycerol, 2.5mM EDTA, pH7.6 supplemented with Complete Roche Proteinase Inhibitor; Roche). Protein concentration was measured by protein assay kit (Bio-Rad Laboratories, Hertfordshire, UK) according to the manufacturer's protocol. Proteins were separated by electrophoresis on 10% SDS-PAGE gels (Invitrogen), transferred onto PVDF membranes, blocked in 5% fat-free milk then incubated with antibodies raised against 11βHSD1 (in house sheep antibody) (25) or αSMA (Abcam, Cambridge, UK) overnight at $4^{o}C$ and a fluorescent secondary antibody. Visualization of antibodies was performed with the Odyssey Clx Imaging system (Li-COR, Cambridge, UK). Pixels were quantified in Photoshop CS3. Protein levels were corrected for GAPDH (Millipore, Hertfordshire, UK) levels.

RNA extraction and real time qPCR. Livers or hepatic stellate cells (HSCs) were homogenized with Qiazol and RNA extracted by RNeasy Mini kit (tissues) or RNeasy Micro kit (cells) according to the manufacturer's protocol (Qiagen, Manchester, UK). The mRNA concentration was measured by Nano-drop spectrophotometer (NanoDrop ND-100,Fisher, Loughborough, UK). Superscript III (Invitrogen) was used for cDNA synthesis and real time PCR was performed using a Roche lightcycler 480 and the UPL probe system (Universal Probe Library, Roche Diagnostics, Burges Hill, UK) with in-house-designed primers (primer/probe combinations are listed in Supporting Table 1). 18S RNA was used as a reference gene to normalize target genes.

In situ hybridization. A plasmid encoding part of the mouse *Hsd11b1* cDNA (26) was linearized with *BamH1* or *Hind III* to generate templates for sense or antisense RNA synthesis, respectively. ^{35}S-radiolabelled sense and antisense probes were prepared as described (26) with SP6 and T7 enzymes, respectively. Probes were purified by column (Amersham Biosciences, Buckinghamshire, UK) according to the manufacturer's protocol then incubated with frozen sections at $55^{o}C$ overnight. Following hybridisation, sections were washed and air dried then subject to autoradiography (FujiFilm, Bedford, UK). For detailed visualisation of anti-sense probe hybridisation, sections were dipped in photographic emulsion (Kodak) and exposed for 4 weeks. Following development in D19 developing

solution (Kodak), sections were counter stained with haemotoxylin and eosin and silver grains quantified using image analysis KS300 (Zeiss, Cambridge, UK).

11βHSD1 activity assay in liver and HSCs. 11β-HSD1 activity was measured in whole liver lysates in the dehydrogenase direction as previously described (27). Liver samples were homogenized in C buffer (30mM NaCl, 1mM EDTA, 50mM Tris, 12% glycerol pH7.7). 0.2 mg/ml protein was added to ^{3}H- corticosterone (10nM) and cofactor NADP (400μM). Steroids were extracted by ethyl acetate and reconstituted in mobile phase solution (60% water, 25% methanol and 15% acetonitrile) ^{3}H-11dehydro-corticosterone and ^{3}H-corticosterone peaks were analysed by HPLC with on-line scintillation counting (ASI 100). 11β-HSD1 activity in cultured HSCs was measured in the reductase direction. [^{3}H]-11-dehydrocorticosterone was prepared by incubating rat placental homogenate with ^{3}H-corticosterone (Amersham Biosciences, Buckinghamshire, UK) and cofactor NAD ^{3}H-11dehydro-corticosterone was added to cells and incubated for 24 hours. Steroids were extracted from the culture medium using C18 reverse-phase Sep-Pak columns (Waters, Elstree, UK) according to the manufacturer's protocol and were analysed by HPLC with online scintillation detection.

Supplemental Table 1. Primers used for qPCR

Gene Name	Forward primer	Backward primer	Probe #
18S	ctcaacacgggaaacctcac	cgctccaccaactaagaacg	77
COL-3	tcccctggaatctgtgaatc	tgagtcgaattggggagaat	49
	catgttcagctttgtggacct	gcagctgacttcagggatgt	15
MMP-2	taacctggatgccgtcgt	ttcaggtaataagcacccttgaa	77
MMP-9	acgacatagacggcatcca	gctgtggttcagttgtggtg	19
MMP-12	ttgtggataaacactactggaggt	aaatcagcttggggtaagca	51
MMP-13	gccagaacttcccaaccat	tcagagcccagaattttctcc	89
TIMP-1	tcagagcccagaattttctcc	agggatagataaacagggaaacact	76
MCP-1(CCL-2)	ctcttgagcttggtgacaaaaa	ggctggagagctacaagagg	62
TGF-β1	tggagcaacatgtggaactc	cagcagccggttaccaag	72
IL-1	tgtaatgaaagacggcacacc	tcttctttgggtattgcttgg	78
CXCL-1	agactccagccacactccaa	tgacagcgcagctcattg	83
11β-HSD1	tctacaaatgaagagttcagaccag	gcccgagtacaatcacttt	1
GR	caaagattgcaggtatcctatgaa	cttggctcttcagaccttcc	91
GILZ	tccgttaaactggataacagtgc	tggttcttcacgaggtccat	49
αSMA	actctcttccagccatctttca	ataggtggtttcgtggatgc	58
YM-1	n/a ABI assay NM_009892.2		
B-Actin	n/a ABI assay NM_007393.3		

Supporting Figure 1

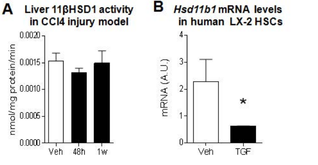

Supporting Figure 2

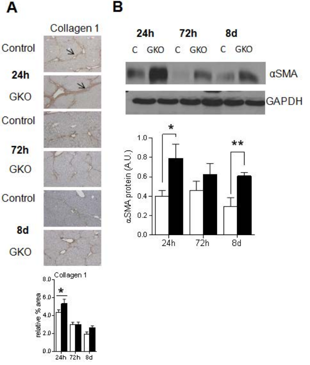

Supporting Figure 3

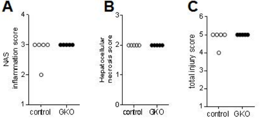

Inflammation, foci/20x field	
None	0
<2 foci	1
2-4 foci	2
>4 foci	3

Hepatocellular necrosis	
None	0
Single cell	1
Confluent	2
Zonal	3
Panacinar	4

Supporting Figure 4

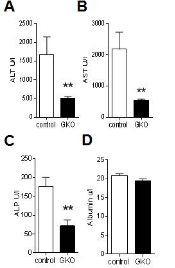

Supporting Figure 5

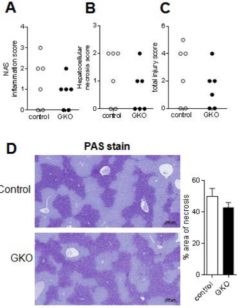

Supporting Figure 6

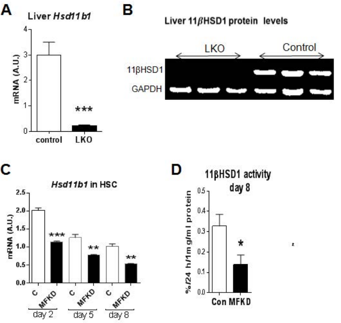

Supporting Figure 7

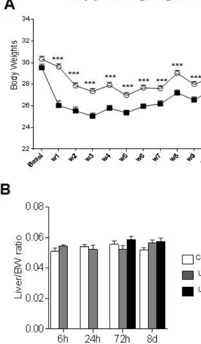

Supplement: Supplementary file 1 — Supporting Information 1 [file HEP-67-2167-s001.pdf]
