# Supplementary material for: 11Beta‐hydroxysteroid dehydrogenase‐1 deficiency or inhibition enhances hepatic myofibroblast activation in murine liver fibrosis
Source: Hepatology. 2018 Feb 22;67(6):2167–81. doi: 10.1002/hep.29734 (PMC6001805; doi:10.1002/hep.29734)
Supplement: Supplementary file 2 — Supporting Information Figure Caption [file HEP-67-2167-s002.docx]

**Supporting Fig.S1:**  **11βHSD1 levels in CCl_4_ liver injury and in human HSCs in culture.**

(A) 11βHSD1 activity in liver homogenates from the CCl_4_ injury model (n=6/group) after vehicle or after 48 hours or 1 week of CCl_4_ administration. (B) *Hsd11b1* mRNA levels were measured in human LX-2 HSC incubated in serum-free medium (n=3 replicates per treatment) and treated with vehicle or activated with TGF-β (2ng/ml) for 16h. * p<0.05 tested by one-way ANOVA (A) or T-test (B).

**Supporting Fig.S2:** **Higher hepatic collagen 1 and αSMA protein levels in GKO in the CCl_4_ injury model**. . (A) Representative images and quantification graph of collagen 1 staining during injury (24h) and resolution (72h and 8 days) phases, with arrowheads pointing to intense collagen 1 staining in control (white bars) and GKO (black bars) mice. (B) Representative hepatic α-SMA western blot image and quantification graph in control and GKO mice at peak (24h) and resolution (72h, 8d) time points in the CCl_4_ liver fibrosis model, n=6/group. * p<0.05; ** p<0.01 between groups tested by two-way ANOVA.

**Supporting Fig.S3: Similar histopathology scores in GKO and control mice in chronic CCl4 injury model.** GKO (black circles) and control (open circles) liver H&E stained sections from the 24h peak fibrosis time point were scored for (A) NAS inflammation (insert table showing the scoring system) (B) hepatocellular necrosis (insert table showing the scoring system) and (C) total liver injury was calculated by adding the scores from A and B. n=6/group. There was no ballooning or steatosis or Mallory bodies observed.

**Supporting Fig.S4: GKO mice show lower plasma transaminases after acute single dose CCl4 injury compared to control mice.** Plasma levels of alanine (ALT; A), aspartate (AST; B) aminotransferases and alkaline phosphatase (ALP; C) and albumin (D). n=6/group; * p<0.05; ** p<0.01 between genotypes tested with Student’s t test.

**Supporting Fig.S5:** **GKO mice have similar hepatocellular damage and necrotic areas after single dose CCL4 injury compared to control mice**. GKO (black circles) and control (open circles) liver H&E stained sections from a single CCl4 dose were scored for (A) NAS inflammation (B) hepatocellular necrosis and (C) total liver injury was calculated by adding the scores from A and B. n=6/group. There was no ballooning or steatosis or Mallory bodies observed. (D)Representative images (scale bar 200µm) and quantification graph of Periodic acid-Schiff (PAS) stain in livers of control (white bars) and GKO mice (black bars). Pale blue stain indicates necrotic areas.

**Supporting Fig.S6:** **Recombination efficiency in LKO and MFKD mouse models**.**(A)** Hepatic *Hsd11b1* mRNA normalized for 18S and (B) 11β-HSD1 protein normalized for GAPDH in LKO mice shows almost complete 11β-HSD1 ablation compared to control (*Cre-*) littermates (n=6/group). *** p<0.001 between groups tested with Student’s t-test. (C) *Hsd11b1* mRNA normalized for 18S in isolated HSCs from MFKD (black bars)and control littermates ( white bars) during 2-5-8 days post *ex-vivo* HSC activation (n=3/group). For clarity only significant differences ** p<0.01; *** p<0.001 * between genotypes within a time point are shown. Note that as seen if Fig. 2B *Hsd11b1* mRNA is reduced during HSC activation in control mice. (D) 11βHSD1 enzymatic activity (% conversion 11-dehydrocorticosterone to corticosterone) was measured at day 8 post HSC activation (n=5/group).

**Supporting Fig.S7: Body and liver weights after UE2316 inhibitor administration.**  (A) body weight (BW) comparisons between chow diet (open circle) and UE2316 (black square) groups during the 12 week CCl4 injury model (B) liver weights corrected for BW between chow diet (white bars) and UE2316 (grey bars) and UE-R (black bars) groups during the 12 week CCl4 injury model. Repeated measures ANOVA, ** p<0.01; *** p<0.001 significant differences between groups.
